# Surface and Structural Properties of Medical Acrylonitrile Butadiene Styrene Modified with Silver Nanoparticles

**DOI:** 10.3390/polym12010197

**Published:** 2020-01-12

**Authors:** Magdalena Ziąbka, Michał Dziadek, Kinga Pielichowska

**Affiliations:** 1 Department of Ceramics and Refractories, Faculty of Materials Science and Ceramics, AGH University of Science and Technology, 30-059 Krakow, Poland; 2 Department of Glass Technology and Amorphous Coatings, Faculty of Materials Science and Ceramics, AGH University of Science and Technology, 30-059 Krakow, Poland; dziadek@agh.edu.pl; 3 Faculty of Chemistry, Jagiellonian University, 30-387 Krakow, Poland; 4 Department of Biomaterials and Composites, Faculty of Materials Science and Ceramics, AGH University of Science and Technology, 30-059 Krakow, Poland; kingapie@agh.edu.pl

**Keywords:** acrylonitrile butadiene styrene, silver nanoparticles, composites, physicochemical properties

## Abstract

Acrylonitrile butadiene styrene/silver nanoparticles (ABS/AgNPs) composites were manufactured through the plastic processing method. Three different matrices were used to obtain polymer and composite samples containing 0.5 wt % and 1.0 wt % of silver nanoparticles, respectively. The aim of this study was to examine physicochemical properties and stability of the materials in the in vitro conditions for two years. The results showed that composites made from amorphous matrices had comparable mechanical properties after incorporation of AgNPs. The values of Young modulus and tensile strength increased after the first and second year of investigation. Silver nanoparticles did not alter the surface parameters—e.g., roughness and contact angle also retained stable values after the in vitro incubation in water solution. The scanning electron observation revealed homogeneous distribution of silver modifier in all the matrices. The 24-month incubation of materials proved the stability of the composites microstructure. The DSC analysis revealed that addition of AgNPs may decrease glass transition temperature of the composite materials which was also reduced after 12 and 24 months of incubation. The attenuated total reflectance–Fourier transform infrared (ATR-FTIR) spectroscopic studies did not indicate significant changes in the ABS matrices either upon their modification with AgNPs or after the long-term testing. The conducted studies proved that all the composites are stable and may be used for a long-term working period.

## 1. Introduction

Acrylonitrile butadiene styrene is an impact-resistant engineering thermoplastic and amorphous polymer. It is made of three monomers: acrylonitrile, butadiene, and styrene. It is endowed with several desirable physical properties, such as high rigidity, good impact resistance even at low temperatures, good insulating properties, good weldability, and abrasion and strain resistance [1,2]. Apart from these characteristics, ABS is also mechanically strong, durable and stable over time. The polymer is not only chemically resistant but it also exhibits high surface brightness and excellent surface aspect. The high impact strength and heat performance make it suitable for use in automotive, electronics, building and construction, home appliances, and transportation industries [3,4]. ABS also offers a variety of possibilities in the medical industry sectors thanks to its exceptional purity, low residual monomers, lot-to-lot consistency, and superior whiteness. On the medical market ABS resin is applied to manufacture, e.g., respiratory devices (dry powder inhaler and metered dose inhaler), infusion systems (connectors, caps, spikes, slide clamps, and roller clamps), auto-injection devices (lancing devices, insulin pen, and auto-injection pens), and others (housing for medical devices and closed system transfer device) [5]. ABS polymer is also a very promising material to produce miniature implants, such as middle ear prostheses [6,7,8]. The long-term implant placement in the human body requires an extremely careful selection of the material and design. The requirements can be classified into four main categories, including the chemical, mechanical, electrical, and thermal characteristics. The properties of polymer materials for medical non-removal implants have to mimic the structural and mechanical characteristics of the tissue they are to replace. First, the implanted polymer devices should maintain their operational capabilities within the biological environment of the body [9]. Second, biostability must be a key factor while choosing a polymer for any biomedical use [10]. From the biological perspective materials should be bioinert and biocompatible [11,12]. To enhance desirable antibacterial properties, such as resistance to microbial colonization and pathogenic microorganism spreading, polymers can be additionally modified with silver, gold, zinc and copper nanoparticles [13,14,15,16,17]. However, the nanoparticles incorporation affects the structure and degradation mechanism of polymer matrix and the resulting physical properties of the material [18]. Such changes may lead to the wear debris of nondegradable implants which then require the surgical removal [19]. On the other hand, implants failures are common due to bacterial infection and biofilm formation. This mainly concerns transcutaneous implants, such as dental implants, catheters or external fixators [20]. Therefore, it is important to prevent the bacterial growth by using antibiotic therapy or by modifying implants by introducing antibacterial additives.

The introduction of various modifying additives to ABS may also change the mechanical properties. Torrado A.R. et al. [21] performed an analysis of the impact of various additives, such as jute plant fibre, titanium dioxide (TiO_2_), zinc oxide (ZnO) nanorods, strontium titanate (SrTiO_3_), and alumina (Al_2_O_3_) on mechanical property anisotropy. Parameters such as: tensile strength and elongation at break were measured and the composites microstructure was evaluated. Authors proved that some of additives improved mechanical properties of composites. They also revealed a lower complex viscosity above *Tg* would improve anisotropy by creating a stronger layer to layer bond and increased surface area contact between layers. Al-Hydary et al. [22] proved that the addition of anatase nanoparticles into the ABS matrix improves the stiffness and the compressive strength of the terpolymer with a noticeable reduction in the flexural strength due to the development of minor defects in the structure. They also found that 10 wt % of anatase nanoparticles is suitable to obtain the best combination between the mechanical properties of composites and their high resistance to bacterial adhesion. Although there are some studies on the modification of ABS with different additives, there is a gap in the literature on their impact on the long-term stability of polymers.

This paper summarizes the production process and examinations of composites from three different ABS matrices, modified with different silver nanoparticles contents. Our aim was to prove that a low concentration of silver nanoparticles does not necessarily change all the physicochemical properties of amorphous polymers. To evaluate the stability of materials during the long-lasting performance we carried out the SEM, DSC, TG, and FTIR examinations during the in vitro incubation for 12 and 24 months. We also used a tensile tester to investigate the mechanical properties of the composite materials and evaluated the surface properties.

## 2. Materials and Methods

### 2.1. Material Manufacturing

The samples for our studies were shaped as paddles. To obtain the samples we used three commercially available polymers ABS Acrylonitrile Butadiene Styrene - Novodur HD15 (INEOS Styrolution, Frankfurt, Germany), marked as HD15 (*MVR* = 15 cm^3^ 10 min^−1^, *E_t_* = 2300 MPa), ElixM (Elix Polymers, Spain) marked as E205FC (*MVR* = 20 cm^3^ 10 min^−1^, *E_t_* = 2550 MPa) and E203FC (*MVR* = 31 cm^3^ 10 min^−1^, *E_t_* = 2400 MPa). We prepared the composite materials modified with 0.5 and 1.0% wt nanosilver (NanoAmor, Katy, TX, USA), 99.9% purity, particle size of 80 nm, and density of 10.49 g cm^−3^. Polymer matrices were already tinned with titanium dioxide by their manufacturers. The plastics processing methods: extrusion and injection molding led to obtaining polymer and composite materials. During the first stage of the process, the granulates were prepared and dried in the laboratory dryer at 80 °C for 4 h. Then, the nanosilver particles were added to the polymer granulates and homogenized in the plasticizing chamber (length of the screw −0.8 m; homogenization temperature: 240 °C). Next, the obtained material was injected into the steel molding form, cooled and extracted. The injection temperature in three zones was 240 °C, the injection pressure −80 kg cm^−2^, and flow: 80%; the above parameters were chosen in accordance with the characteristic data sheet of polymer manufacturer.

### 2.2. Material Evaluation

The materials were immersed in deionized water at 37 ± 1 °C for 12 and 24 months in tightly closed, sterile polypropylene containers. The sample weight to incubation medium ratio was 1 g:10 mL, which complied with the ISO 10993-13:2010 standard: Identification and quantification of degradation products from polymeric medical devices [23]. The examinations were performed both before and after the incubation.

#### 2.2.1. Scanning Electron Microscopy

The detailed microstructure examination of the materials was carried out by means of the Nova NanoSEM 200 scanning electron microscope (FEI, Eindhoven, The Netherlands) with an Genesis XM X-ray microanalysis system (EDAX, Tilburg, The Netherlands) featuring the EDAX Sapphire Si(Li) EDX detector. The samples were coated with a carbon layer. The observations and measurements took place in high vacuum conditions, using a backscatter electron detector (BSE) with the accelerated voltage of 10–18 kV. The distribution of elements was recorded in mapping and spectrum analysis.

#### 2.2.2. Roughness

We evaluated the arithmetical mean roughness (*Ra*) of the HD15, E205FC, E203FC and their nanosilver composites by means of a contact profilometer HOMMEL-ETAMIC T1000 wave (Jenoptik AG, Jena, Germany). The arithmetical mean roughness values were an average of 10 measurements expressed as the mean ± standard deviation (SD).

#### 2.2.3. Surface Wettability

The static water contact angle was engaged to assess the surface wettability using the sessile drop method with an automatic drop shape analysis system DSA 10 Mk2 (Kruss GmbH, Hamburg, Germany). Constant temperature and humidity conditions were maintained throughout the tests while UHQ-water droplets of 0.25 μL were applied on each pure and dry sample. We calculated the apparent contact angle as an average of 10 measurements and expressed it as a mean ± standard deviation (SD).

#### 2.2.4. Tensile Test

To evaluate the tensile strength (*σ_M_*) and Young modulus (*E_t_*) a universal testing machine Inspect Table Blue 5 kN with 5 kN load cell (Hegewald&Peschke, Nossen, Germany) was used. The assumed value of pre-load force was 1 N and the test speed −50 mm min^−1^. The samples examinations followed the EN ISO 527-1 standards [24]. The mechanical parameters were obtained after averaging 10 measurements and expressed as a mean ± standard deviation (SD).

#### 2.2.5. Differential Scanning Calorimetry and Thermogravimetry

The DSC and TG examinations were conducted using the Netzsch STA 449F3 Jupiter (Netzsch-Gerätebau GmbH, Selb, Germany). The samples weighing ca. 4 mg were placed in sealed aluminum pans. We selected the heating rate to be 10 °C min^−1^ and used argon as an inert gas (the flow rate −40 mL·min^−1^). Beforehand, an empty aluminum pan served as a reference and the calorimeter was calibrated with aindium standard. The glass transition temperature (*T_g_*) was calculated as the inflection point of the first derivative from the DSC curves.

#### 2.2.6. Attenuated Total Reflectance Fourier Transform Infrared Spectroscopy

The attenuated total reflectance–Fourier transform infrared (ATR-FTIR) analysis was conducted by means of the VERTEX 70V spectrometer (Bruker, Billerica, MA, USA). The spectra were registered in the 550 to 4000 cm^−1^ wavenumber range using a platinum single crystal diamond ATR unit. In total, we accumulated 128 scans at 4 cm^−1^ resolution.

#### 2.2.7. Statistical Analysis

The results were analyzed using one-way analysis of variance (ANOVA) with Duncan post-hoc tests, which were performed with Statistica 13.1 software (TIBCO Software Inc., Palo Alto, CA, USA). The results were considered statistically significant when *p* < 0.05.

## 3. Results

**Figure 1:**
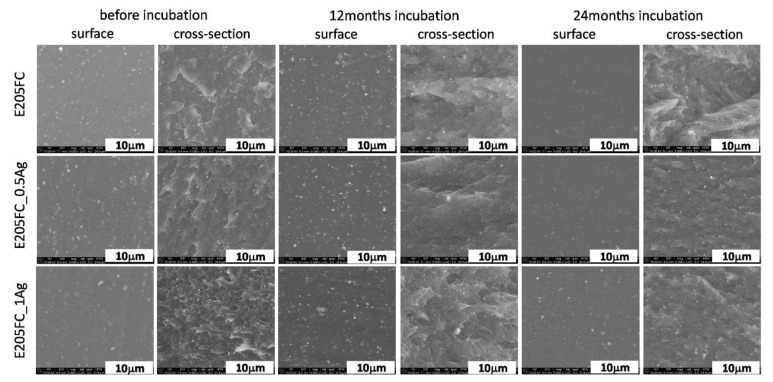
SEM images of surface and cross-section of pure polymer (E205FC) and polymer containing 0.5 wt % and 1.0 wt % of silver nanoparticles AgNPs before and after 12 and 24 months of incubation.

**Figure 2:**
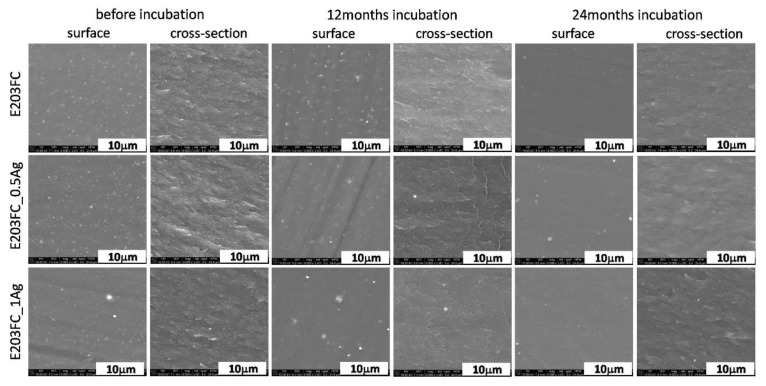
SEM images of surface and cross-section of pure polymer (E205FC) and polymer containing 0.5 wt % and 1.0 wt % of silver nanoparticles AgNPs before and after 12 and 24 months of incubation.

**Figure 3:**
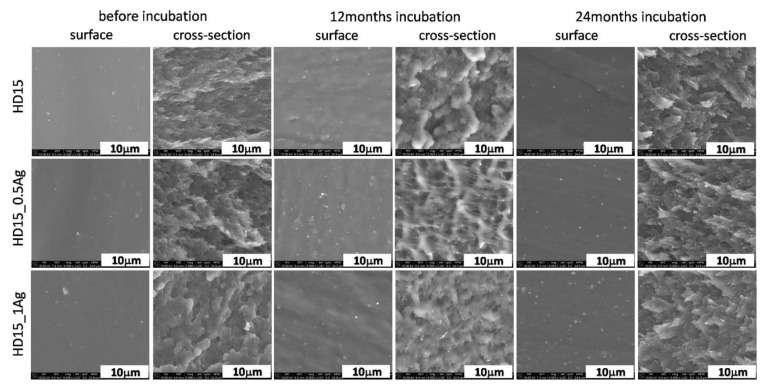
SEM images of surface and cross-section of pure polymer (HD15) and polymer containing 0.5 wt % and 1.0 wt % of silver nanoparticles AgNPs before and after 12 and 24 months of incubation.

The microscopic tests of polymers E205FC (Figure 1), E203FC (Figure 2) and HD15 (Figure 3) and their silver composites revealed that metal nanoparticles were homogeneously dispersed throughout the matrix volume. Additionally, the surfaces of the tested materials were homogeneous and smooth, without any visible agglomerates. However, individual silver agglomerates appeared both on the surface of the materials and in cross-sections of the composites with 1.0 wt % of AgNPs. No significant changes were noticed in the surface studies after 12 and 24 months of incubation in the water solution, which was also proved by mapping analysis (Figure S1 of the Supplementary Materials). However, a lower content of nanoparticles was noticed on the surface investigated after 24 months of incubation. This characteristic behavior was observed for all the tested polymers and may also be associated with the release of Ag^+^ ions from silver nanoparticles and leaching of the nanoparticles themselves from the polymer matrix, which was confirmed in previous works by the authors [7,8]. In the cross-sections of polymers and composites, we did not observe any cracks indicating structural changes in polymer matrices, which proved the low possibility of their degradation. An equally high degree of AgNPs homogeneity was also noted for the composites with the 0.5 wt % silver content. The cross-section observations revealed the presence of silver nanoparticle agglomerates. Due to further analysis we concluded that areas of silver nanoparticles were more common for the composites with 1.0 wt % AgNPs. As for the observations after 12 and 24 months of incubation, the microstructure of polymers and composites did not change. Both the samples surface and cross-sections displayed areas with very uniform dispersion of nanoparticles and a few agglomerates of nanoparticles, visible only for composites with 1.0 wt % of silver.

**Figure 4:**
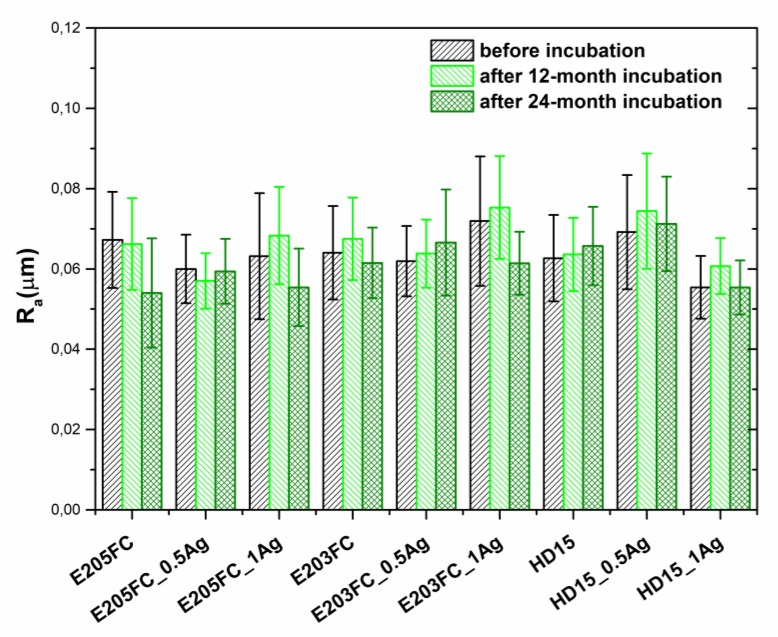
The arithmetical mean roughness (*Ra*) of pure polymers and polymers containing 0.5 wt % and 1.0 wt % of silver nanoparticles AgNPs. Statistical analysis showed no differences (*p* < 0.05) between materials before and after 12- and 24-month incubation.

The roughness tests (Figure 4) showed no significant changes associated with the silver nanoparticles presence in the matrices. For all the three polymers, the roughness values were in the range between 0.063 µm and 0.067 µm. In the case of 0.5 wt % AgNPs composites the *Ra* parameter was between 0.060 µm and 0.069 µm, while for the AgNPs 1.0 wt % composites—between 0.056 µm and 0.072 µm. The study conducted after 12 and 24 months of incubation did not display any statistically significant changes (*p* < 0.05). After one year, the roughness value was in the range of 0.064 µm to 0.068 in the case of pure polymers, 0.057 µm to 0.075 µm for the composites with 0.5 wt % AgNPs and 0.061 µm to 0.075 µm for the ones with 1 wt % AgNPs. After two years of incubation, the roughness values for the pure polymers ranged from 0.054 µm to 0.066 µm, for the composites with 0.5 wt % AgNPs—0.060 µm to 0.071 µm—and for the composites with 1 wt % AgNPs—0.055 µm to 0.061 µm—respectively.

**Figure 5:**
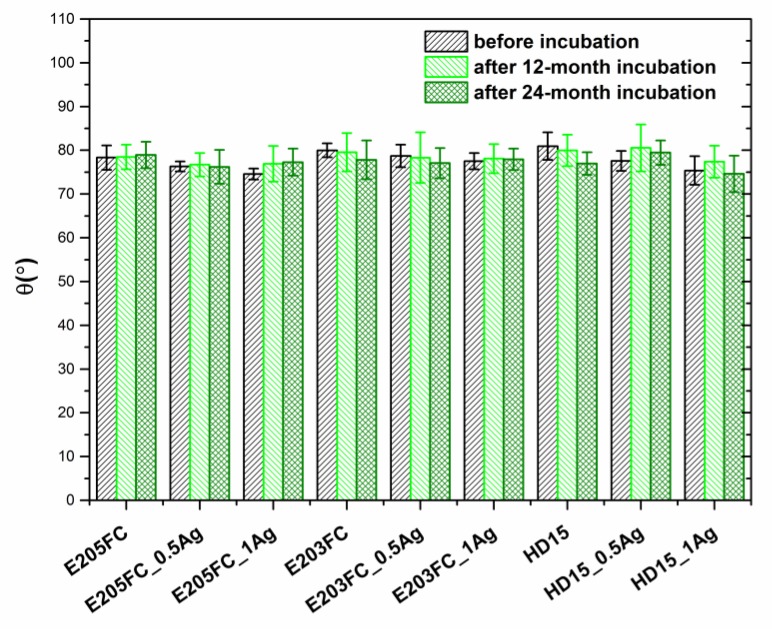
Static water contact angle of pure polymers and polymers containing 0.5 wt % and 1.0 wt % of silver nanoparticles AgNPs. Statistical analysis showed no differences (*p* < 0.05) between materials before and after 12- and 24-month incubation.

The contact angle analysis carried out for pure polymers and their nanosilver composites showed no change in the values, regardless of the modifying phase proportions (Figure 5). The contact angle equaled 78° for E205FC and 80° for E203FC and HD15 for the pure polymer matrices. These values did not alter significantly after 12- and 24-month incubation in deionized water (*p* < 0.05). The values of contact angle for the composites containing 0.5 wt % AgNPs before the incubation were 76° for E205FC and 78° for E203FC and HD15. In the case of the composites containing 1.0 wt % AgNPs, the angle values were: 75° for E205FC and HD15, and 78° for E203FC, respectively. The tests performed after 12 and 24 months of incubation confirmed the stable contact angle values for all the tested materials.

**Figure 6:**
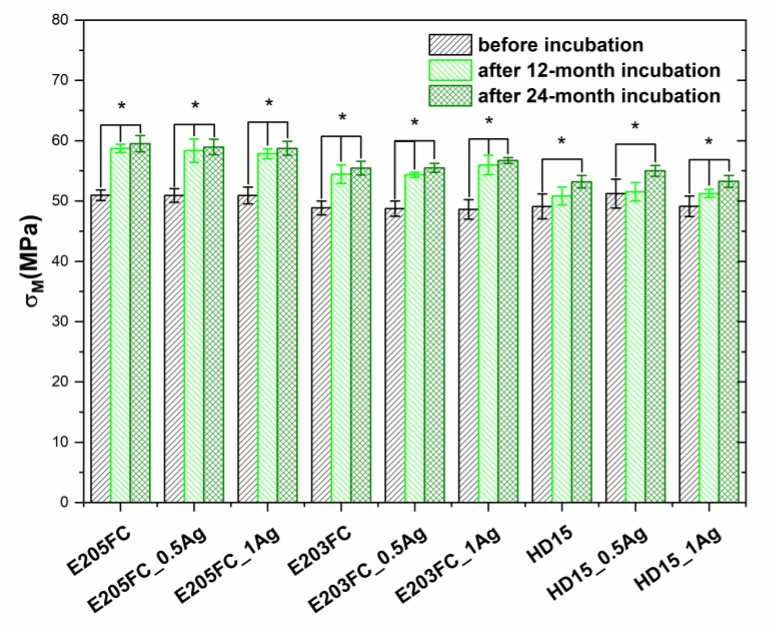
Tensile strength (*σ_M_*) of pure polymers and polymers containing 0.5 wt % and 1.0 wt % of silver nanoparticles AgNPs. Statistically significant differences (*p* < 0.05) between materials before and after 12- and 24-month incubation are indicated by asterisk (*).

Introduction of silver nanoparticles in the amount of 0.5 wt % and 1.0 wt % into the polymer matrices did not affect their mechanical properties (Figure 6, Table 1). For all the tested samples, basing on E205FC and HD15, the average value of tensile strength was in the range of 49.1 to 51.2 MPa. For the samples on the E203FC matrix it was slightly lower—in the range of 48.6 to 48.9 MPa. However, the examinations conducted after 12 and 24 months of incubation showed an increase in the samples strength. The changes were statistically significant, especially those after 12 months of incubation (*p* < 0.05). The materials based on E205FC and E203FC matrices revealed the largest increase after 12 months of incubation. In the case of the HD15 materials, a gradual increase in the value of Young modulus was observed throughout the incubation period.

**Figure 7:**
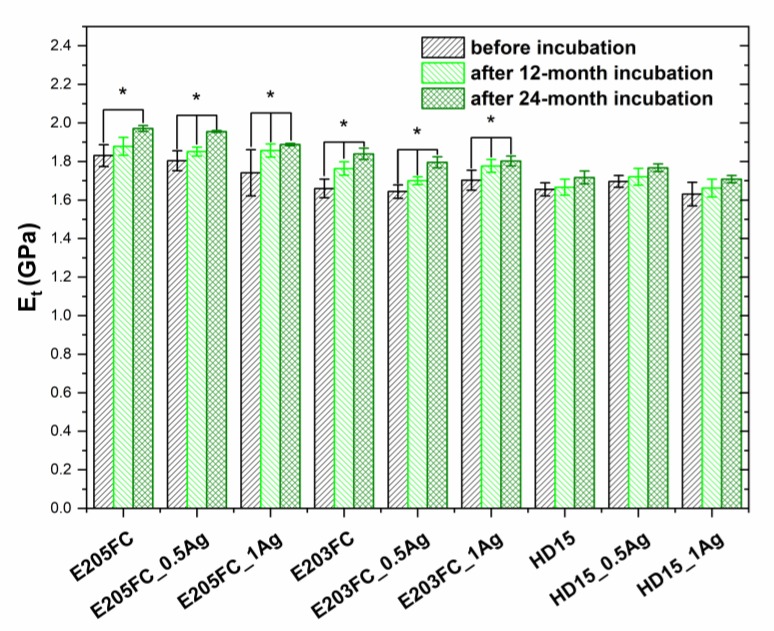
Young modulus (*E_t_*) of pure polymers and polymers containing 0.5 wt % and 1.0 wt % of silver nanoparticles AgNPs. Statistically significant differences (*p* < 0.05) between materials before and after 12- and 24-month incubation are indicated by asterisk (*).

The Young modulus values for all the materials was determined via mechanical tests (Figure 7, Table 1). For the E205FC matrix and E205FC_0.5Ag and E205FC_1Ag composites, the average value was in the range of 1.74–1.83 GPa. For the E203FC, E203FC_0.5Ag, and E203FC_1Ag matrices, the HD15 matrix, and HD15_0.5Ag and HD15_1Ag composites, the average value was 1.63–1.7 GPa. There were no changes in the Young modulus noted with the increasing share of the modifying phase. However, a gradual increase in the values was observed during the entire incubation period for all the tested polymers and their composites containing silver nanoparticles. What is more, the results obtained for E205FC-based and E203FC-based materials were statistically significant, especially those after 12 months of incubation (*p* < 0.05).

**Figure 8:**
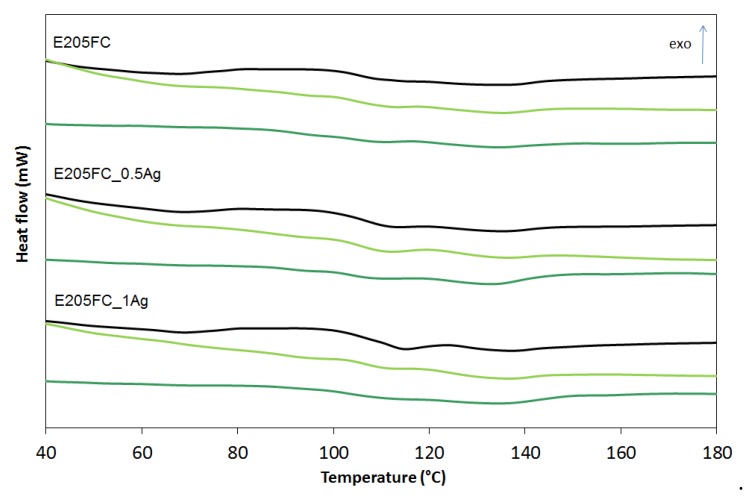
Glass transition temperature (*T_g_*) of pure polymer (E205FC) and polymer containing 0.5 wt % and 1.0 wt % of silver nanoparticles AgNPs before incubation (black line), after 12 months (light green line) and after 24 months of incubation (dark green line).

**Figure 9:**
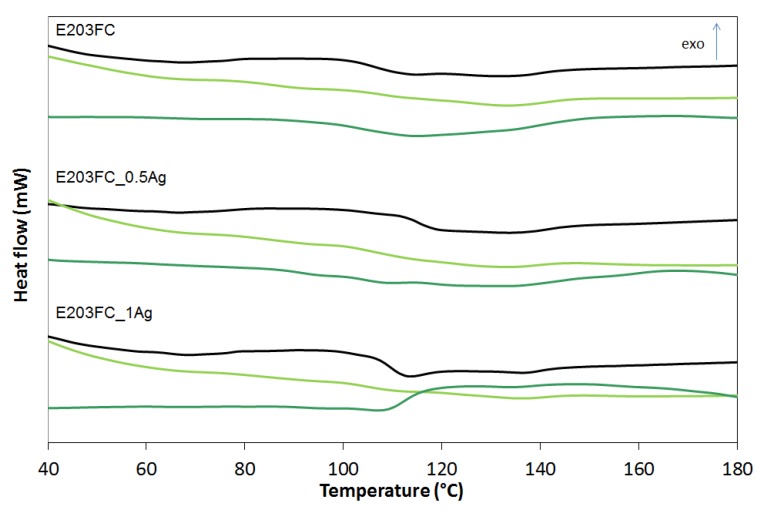
Glass transition temperature (*T_g_*) of pure polymer (E203FC) and polymer containing 0.5 wt % and 1.0 wt % of silver nanoparticles AgNPs before incubation (black line), after 12 months (light green line) and after 24 months of incubation (dark green line).

**Figure 10:**
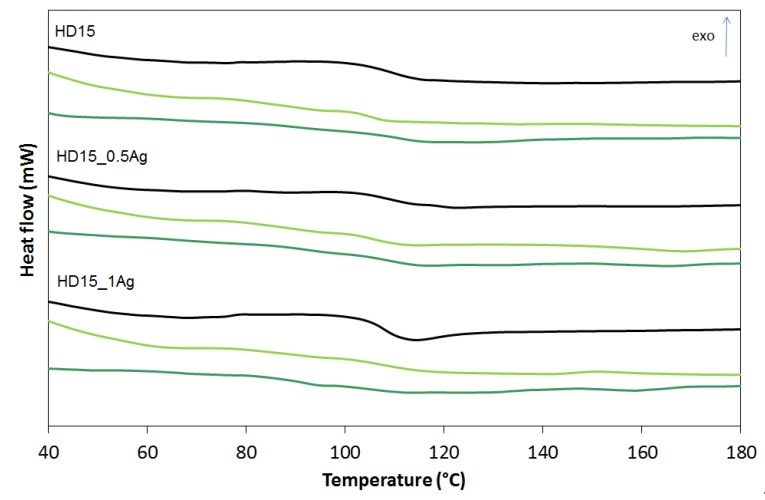
Glass transition temperature (*T_g_*) of pure polymer (HD15) and polymer containing 0.5 wt % and 1.0 wt % of silver nanoparticles AgNPs before incubation (black line), after 12 months (light green line) and after 24 months of incubation (dark green line).

The differential scanning calorimetry (DSC) examined how the nanosilver introduction and the incubation time influenced the thermal properties of ABS terpolymers. The DSC curves are shown in Figure 8, Figure 9 and Figure 10 and the glass transition temperature (*Tg*) values are summarized in Table 1.

The observations revealed that *Tg* is affected by all the three factors: the type of ABS (also its specific flow rate), the amount of nanosilver, and the incubation time. ABS is a terpolymer obtained by polymerization of 1.3-butadiene and copolymerization of acrylonitrile with styrene while grafting the resulting copolymer onto polybutadiene. Therefore, ABS has two glass transitions: the first connected with polybutadiene at the temperature of approximately −82 °C, and the second, derived from acrylonitrile–styrene copolymer grafted on polybutadiene, occurring at a temperature of about 105 °C [25]. The conducted glass transition from acrylonitrile–styrene copolymer was analyzed. We concluded that for the ABS with higher flow rates (E205F and E203FC) the glass transition temperature went up after the silver nanoparticles introduction, which proved the increasing impact strength and stiffening of the material. The opposite trend was observed for the ABS with the lowest melt flow index (highest average molecular weight). In this matrix the introduced silver nanoparticles acted as a plasticizer, weakening the interactions and lowering the glass transition temperature. Note that the materials incubation lowered the glass transition temperature, which was consistent with the effects observed for other polymers. Yang et al. [26] observed a decrease in the glass transition temperature of polyurethanes after incubation in water. They explained this phenomenon by the plasticizing action of water molecules on macromolecules that weakened interactions between chains and increased the activity of segmental macro chain movements. In our research the only exception was the HD15 materials whose glass transition temperature hardly changed after two years of incubation in water. This result could be associated with the lowest melt flow index and the highest average molecular weight of the polymer characterized by the chains entanglement and the resulting limitation of their mobility.

**Figure 11:**
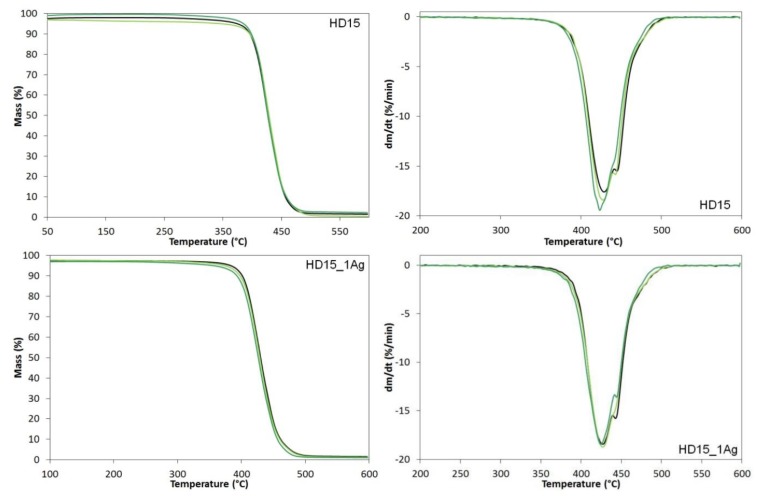
Thermogravimetric (TG) and derivative thermogravimetric (DTG) curves of pure polymer (HD15) and polymer containing 1.0 wt % of silver nanoparticles AgNPs before incubation (black line), after 12 months (light green line) and after 24 months of incubation (dark green line).

The thermogravimetric analysis was performed to establish the thermal stability of the samples. The TG and DTG curves for HD15 and HD15_1Ag samples before and after 12- and 24-month incubation in water are shown in Figure 11. On the basis of the TG curves we established both the thermal stability and the temperatures corresponding to subsequent mass losses (T_1%_, T_3%_, T_5%_, T_10%_,T_50%_), the temperatures corresponding to the highest degradation rates (T_DTGmax_) and the amount of solid residue at 600 °C. The results are presented in Table 2.

In the tests we conducted, all the samples revealed one degradation stage on the TG curves. However, the analysis of the DTG curves indicated that the ABS thermal degradation consisted of at least three overlapping processes. The degradation began at the temperature of about 340–350 °C and happened at the highest rate at ~420–440 °C. In the TG-FTIR study conducted by Jian et al. [27] it was found that butadiene was released mainly in the initial stages of degradation at ~340 °C. At 350 °C, butadiene was still secreted, yet the release of aromatic degradation products also started and the proportion of styrene increased with the volatile decomposition products. The secretion of acrylonitrile began at temperatures above 400 °C and the ammonia formation was observed at about 430 °C.

**Figure 12:**
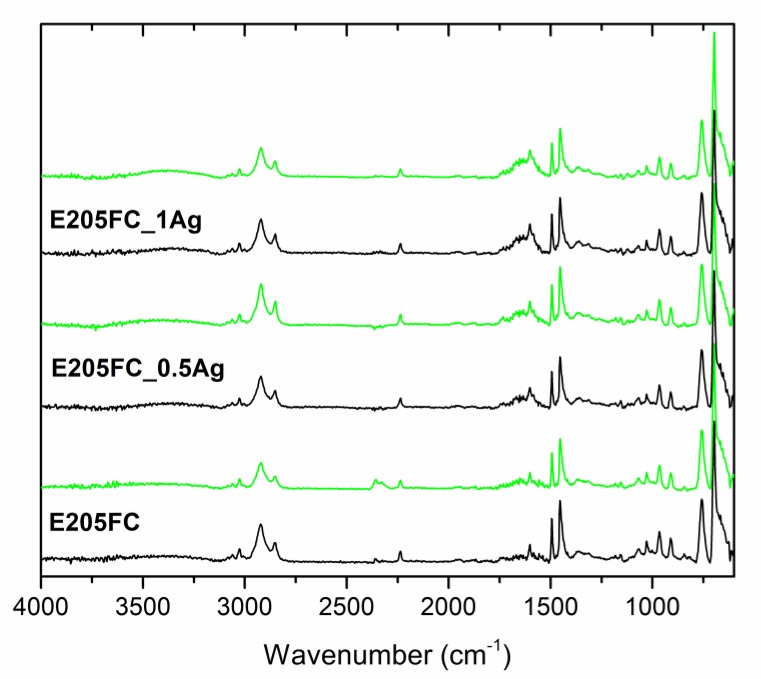
Attenuated total reflectance–Fourier transform infrared (ATR-FTIR) spectra of pure polymer (E205FC) and polymer containing 0.5 wt % and 1.0 wt % of silver nanoparticles AgNPs before incubation (black line) and after 24 months of incubation (light green line).

**Figure 13:**
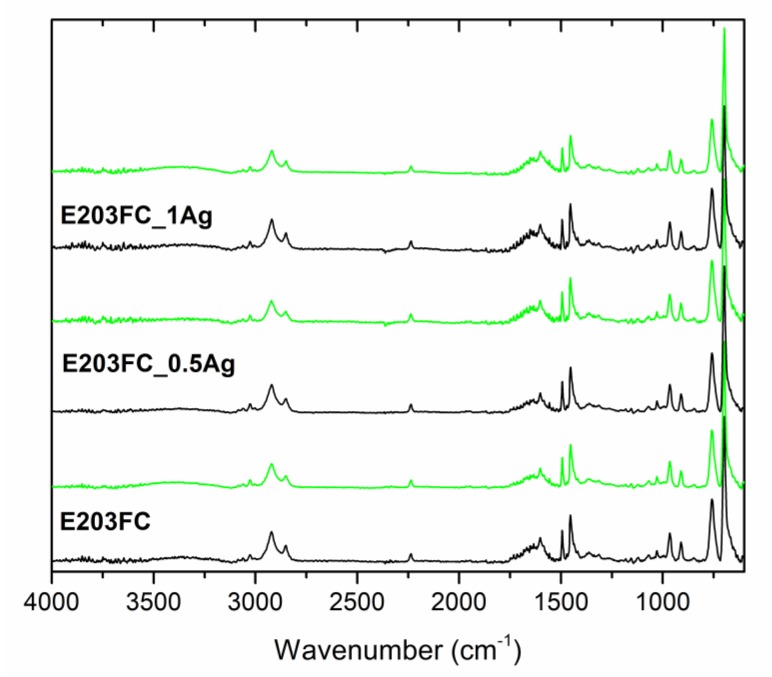
ATR-FTIR spectra of pure polymer (E203FC) and polymer containing 0.5 wt % and 1.0 wt % of silver nanoparticles AgNPs before incubation (black line) and after 24 months of incubation (light green line).

**Figure 14:**
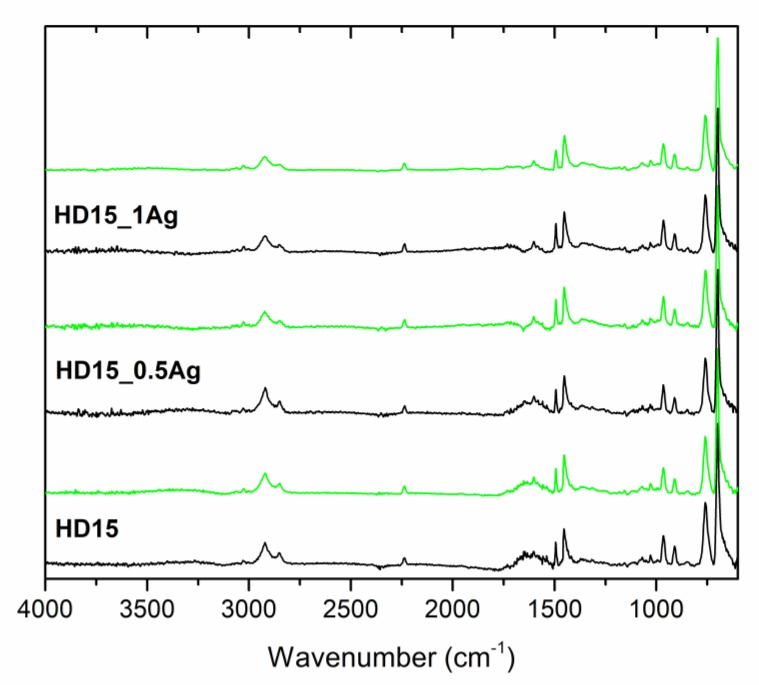
ATR-FTIR spectra of pure polymer (E205FC) and polymer containing 0.5 wt % and 1.0 wt % of silver nanoparticles AgNPs before incubation (black line) and after 24 months of incubation (light green line).

Our structural analysis of pure polymers and polymers containing 0.5 wt % and 1.0 wt % of AgNPs was conducted using the ATR-FTIR technique (Figure 12, Figure 13 and Figure 14, Table 3). The spectra contained bands typical for the ABS polymer, including bands corresponding to individual monomers (acrylonitrile, butadiene and styrene). The obtained results clearly indicated that no changes were observed in the absorption spectra after both AgNPs modification and the 24-month incubation in deionized water.

## 4. Discussion

Modifications of polymer matrices with nanosilver are often aimed at obtaining new antibacterial properties. One of methods to achieve the desired properties is to coat the polymer surface with thin nanolayers. Several studies have been conducted on this topic. In their research Sánchez-Valdes et al. [30] modified polyethylene through the laminating, casting and spraying procedures to deposit antibacterial layers with silver nanoparticles. Zendehnam et al. [31] confirmed that membranes made of ABS coated with an Ag nano-layer showed bactericidal activity against *Escherichia coli*. Avila-Alfaro et al. [32] revealed the biocidal activity of silver nanoparticles deposited on the surface of ABS via a sonochemical process (specifically against the fungus, *Aspergillus niger* and *Eschericha coli*).

Polymer composites based on matrices of amorphous polymers, e.g., ABS modified with silver nanoparticles, could be produced through extrusion and injection methods as well. What is more, they could be endowed with antibacterial properties and high biocompatibility [7,33]. In this paper, we focused on the structural and surface properties of ABS/AgNPs composites tested during 12 and 24 months of water incubation. Based on the conducted research, we proved that the homogeneous dispersion of silver nanoparticles depended on the share of nanoparticles introduced into the matrix. We observed that the 0.5 wt % of AgNPs facilitated the advantageous distribution of nanoparticles in the volume of the composite, while the addition of 1.0 wt % could result in agglomerates presence. As agglomerates acted similarly to impurities as centers of stress concentration in the matrix, they reduced the mechanical properties of the polymer [34]. The incubation in aquatic environment did not change the microstructure of the tested materials. It affected neither the surface parameters nor the parameter of roughness or the contact angle. The number of introduced silver nanoparticles up to the 1.0 wt % share did not change the latter parameters either. On the other hand, the incubation factor was crucial to the materials mechanical properties. After 12 and 24 months of incubation, an increase in both the roughness and contact angle parameters was noted, as compared to the values of initial materials. Based on our previous research on the polypropylene matrices modified with silver nanoparticles, we noticed that the increasing content of silver nanoparticles up to the 1.0 wt. % share did not cause an increase in the composites mechanical parameters. Such an increase was observed only after the incubation [35].

Regarding other works, Ahmed JK et al. [36] showed an increase in the mechanical properties of the ABS-based composites modified with silver nanoparticles (tensile strength and hardness) taking place along with the increasing AgNPs content of 3.0, 6.0, and 9.0 wt %. The composites were formed by dissolving ABS in THF, mixing the solution with AgNPs dispersed in THF and then evaporating the solvent. The researchers explained that the mechanical parameters increased due to additional interactions in the composites that occurred between the active surface of AgNPs and the polar matrix of the ABS polymer, which was indicated by a new band appearing in FTIR studies at the wavenumber of 2358 cm^−1^. They related the polarity of ABS matrix to the presence of nitrile groups in acrylonitrile and concluded that the higher amount of nanoparticles raised the contact angle and lowered the roughness values of the composites.

During our FTIR analyses of all the tested materials such a band did not appear. Moreover, the spectra for pure polymers were similar to those for the composite materials and their character did not change after the 24-month incubation. Such a behavior proved the lack of interaction between AgNPs and polymer matrices and confirmed the stability of the materials. Taking into account our results obtained after two years of incubation, we could undoubtedly conclude that silver nanoparticles introduced into the ABS matrices did not degrade the materials.

Köroğlu A. et al. [37] revealed that the introduction of 0.8% and 1.6% AgNPs into the microwave-polymerized resin significantly decreased the transverse strength and elastic modulus of polymethyl methacrylate (PMMA). The addition of silver nanoparticles did not affect the impact strength, while it reduced the glass transition temperature of polymers. Sodagar A. et al. [38] pointed out the effect of two factors on the mechanical properties of composites. They noticed that the AgNPs influence on flexural strength of PMMA depended on the type of acrylics and the concentration levels of nanoparticles. The same proportion of silver nanoparticles could cause both a decrease and an increase in flexural strength in the PMMA matrix composites obtained from various manufacturers. The reduction in mechanical properties was explained by the low content of AgNPs that acted as impurities and the lack of interaction between the polymer matrix and nanoparticles.

Based on our thermal and thermogravimetric tests for ABS/AgNPs samples, it was noticed that the thermal stability of the polymer matrix increased along with the higher nanosilver share for all the three ABS types. The research results also indicated that the long-term incubation in water lowered the thermal stability of the tested ABS. This could be associated with the water penetrating the material and a higher mobility of macrochains demonstrated in the DSC studies where the TDTGmax was slightly reduced. The slightly bigger decreases in thermal stability were observed for the samples containing silver nanoparticles. This effect could result from the silver ions transition into the solution and the water molecules penetration that facilitated degradation. However, our ATR-FTIR studies did not confirm the degradation of polymer matrices. No changes in thermal stability were observed in the temperature range selected for manufacturing and using the composite materials, even after two years of incubation.

## 5. Conclusions

The methods of injection molding and extrusion of the pure polymer and composite samples were properly selected to obtain well-distributed amorphous matrices via melting and homogenizing in a dual cycle. The best dispersion was achieved for the composites with 0.5 wt % of silver nanoparticles. The concept of incorporating nanosilver into acrylonitrile butadiene styrene matrices did not influence the values of tensile strength and Young modulus. The in vitro tests performed after 12 and 24 months of incubation in deionized water proved the mechanical and microstructural stability of the investigated materials. Both the tensile strength and Young modulus values increased after the incubation. The silver nanoparticles share amounting to 0.5 wt % and 1.0 wt % did not change the surface parameters, such as roughness and wettability. The incubation process itself did not have a significant impact on these parameters either. The DSC measurements revealed the decreasing glass transition temperature along with the incubation time. The changes in the *T_g_* values dependent on the content of silver nanoparticles, the matrix type and their melt flow index were also observed. The ATR-FTIR spectroscopic studies indicated no significant changes upon the modification of ABS matrices with AgNPs or after the incubation periods. Therefore, the conducted studies proved that all tested materials are suitable for a long-term application without the signs of degradation.

## Figures and Tables

**Table 1:** Young modulus, tensile strength, glass transition temperature and specific heat capacity of the pure polymers and polymers containing 0.5 wt % and 1.0 wt % of silver nanoparticles AgNPs.

Sample	Before Incubation				After 12-months Incubation				After 24-Months Incubation			
	Mechanic. Prop.		DSC		Mechanic. Prop.		DSC		Mechanic. Prop.		DSC	
	E_t_ (GPa)	σ_M_ (MPa)	T_g_ (°C)	ΔCp [J/gK]	E_t_ (GPa)	σ_M_ (MPa)	T_g_ (°C)	ΔCp [J/gK]	E_t_ (GPa)	σ_M_ (MPa)	T_g_ (°C)	ΔCp [J/gK]
E205FC	1.83 ± 0.06	51.0 ± 0.9	105	0.260	1.88 ± 0.05	58.7 ± 0.7	104	0.216	1.97 ± 0.02	59.5 ± 1.4	104	0.256
E205FC_0.5Ag	1.80 ± 0.05	50.9 ± 1.1	107	0.384	1.85 ± 0.02	58.4 ± 1.9	105	0.237	1.96 ± 0.01	59.0 ± 1.3	104	0.158
E205FC_1Ag	1.74 ± 0.12	50.9 ± 1.4	112	0.520	1.86 ± 0.03	57.8 ± 0.8	107	0.186	1.89 ± 0.01	58.7 ± 1.2	103	0.144
E203FC	1.66 ± 0.05	48.9 ± 1.2	106	0.324	1.76 ± 0.03	54.4 ± 1.5	107	0.291	1.84 ± 0.03	55.5 ± 1.1	103	0.330
E203FC_0.5Ag	1.64 ± 0.04	48.7 ± 1.3	115	0.365	1.70 ± 0.02	54.3 ± 0.4	105	0.380	1.80 ± 0.03	55.5 ± 0.7	104	0.126
E203FC_1Ag	1.70 ± 0.05	48.6 ± 1.6	109	0.421	1.78 ± 0.03	56.0 ± 1.6	104	0.134	1.80 ± 0.03	56.7 ± 0.5	104	0.053
HD15	1.65 ± 0.03	49.1 ± 2.1	110	0.385	1.67 ± 0.04	50.8 ± 1.5	105	0.196	1.72 ± 0.03	53.2 ± 1.0	111	0.138
HD15_0.5Ag	1.70 ± 0.03	51.2 ± 2.4	109	0.373	1.72 ± 0.04	51.5 ± 1.5	105	0.217	1.78 ± 0.02	55.0 ± 0.9	109	0.146
HD15_1Ag	1.63 ± 0.06	49.1 ± 1.7	108	0.433	1.66 ± 0.05	51.2 ± 0.7	107	0.202	1.71 ± 0.02	53.3 ± 1.0	110	0.154

**Table 2:** Thermal stability of pure polymer (HD15) and polymer containing 1.0 wt % of silver nanoparticles AgNPs before incubation, after 12 months and after 24 months of incubation.

Sample	T_1%_ [°C]	T_3%_ [°C]	T_5%_ [°C]	T_10%_ [°C]	T_50%_ [°C]	T_DTGmax_ [°C]	Char Residue [%]
**After Preparation**							
E205FC	346	380	390	402	430	426.0	9.61
E205FC_0.5Ag	348	381	391	402	428	424.5	2.05
E205FC_1Ag	346	384	392	401	427	423.2	6.29
E203FC	340	377	388	400	429	426.5	2.23
E203FC_0.5Ag	341	380	390	401	430	426.3 445.5	2.25
E203FC_1Ag	348	380	390	402	430	426.6	2.65
HD15	328	376	390	403	432	428.7 444.6	2.14
HD15_0.5Ag	339	382	392	404	431	427.3 443.5	1.92
HD15_1Ag	357	387	396	405	431	427.1 442.8	2.36
**After 12 Months of Incubation**							
E205FC	343	380	390	401	428	421.4	0.51
E205FC_0.5Ag	340	379	390	402	428	424.5	1.59
E205FC_1Ag	333	378	389	402	427	422.1	7.06
E203FC	319	377	389	401	429	426.6	0.11
E203FC_0.5Ag	340	378	389	401	430	428.2	4.06
E203FC_1Ag	336	379	389	401	430	426.6	2.20
HD15	316	376	390	403	431	427.5 442.7	3.59
HD15_0.5Ag	319	378	391	403	431	425.8 443.5	1.87
HD15_1Ag	323	377	390	403	430	427.1	2.66
**After 24 Months of Incubation**							
E205FC	344	368	378	394	422	419.5	5.56
E205FC_0.5Ag	344	366	378	393	422	420.8	9.04
E205FC_1Ag	344	370	382	396	423	420.3	6.37
E203FC	336	371	383	396	426	425.8	2.02
E203FC_0.5Ag	343	373	384	398	427	424.0	0.12
E203FC_1Ag	317	366	381	362	427	424.5	3.26
HD15	324	376	388	400	427	423.3	0.77
HD15_0.5Ag	326	376	388	401	428	424.7	1.47
HD15_1Ag	322	374	387	400	428	424.4 440.0	1.04

**Table 3:** Band positions and their assignments for ATR-FTIR spectra of the pure polymers and polymers containing 0.5 wt % and 1.0 wt % of silver nanoparticles AgNPs, data from [28,29].

Band Position	Band Assignment
3200–3000 cm^−1^	stretching vibrations of aromatic C–H bonds
3000–2800 cm^−1^	stretching vibrations of aliphatic C–H bonds
2237 cm^−1^	stretching vibrations of C≡N bonds in acrylonitrile units
1635 cm^−1^	stretching vibrations of C=C bonds in butadiene units
1600, 1582, and 1492 cm^−1^	stretching vibrations of aromatic ring in styrene units
1452 cm^−1^	scissoring vibrations of CH_2_ groups
967 and 911 cm^−1^	deformation of C–H bonds for hydrogen atoms attached to alkenic carbons in butadiene units
756 and 698 cm^−1^	bending vibrations of C–H bonds in monosubstituted benzene rings

## Supplementary Materials

The following are available online at https://www.mdpi.com/2073-4360/12/1/197/s1, Figure S1: Spectrum and mapping analysis of HD15 polymer containing 1 wt.% of silver nanoparticles (AgNPs) before and 24 months of incubation.
