# Supplementary material for: Surface and Structural Properties of Medical Acrylonitrile Butadiene Styrene Modified with Silver Nanoparticles
Source: Polymers (Basel). 2020 Jan 12;12(1):197. doi: 10.3390/polym12010197 (PMC7023594; doi:10.3390/polym12010197)
Supplement: Supplementary file 1 [file polymers-12-00197-s001.pdf]

polymers

Supplementary Material

Surface and Structural Properties of Medical Acrylonitrile Butadiene Styrene Modified with Silver Nanoparticles

Magdalena Ziąbka [1,*], Michał Dziadek [2,3] and Kinga Pielichowska [4]

1 AGH University of Science and Technology, Faculty of Materials Science and Ceramics, Department of Ceramics and Refractories, Krakow 30-059, Poland
2 AGH University of Science and Technology, Faculty of Materials Science and Ceramics, Department of Glass Technology and Amorphous Coatings, Krakow 30-059, Poland; dziadek@agh.edu.pl
3 Jagiellonian University, Faculty of Chemistry, Krakow 30-387, Poland
4 AGH University of Science and Technology, Faculty of Materials Science and Ceramics, Department of Biomaterials and Composites, Krakow 30-059, Poland; kingapie@agh.edu.pl
* Correspondence: ziabka@agh.edu.pl; Tel.: +48-012-617-2523

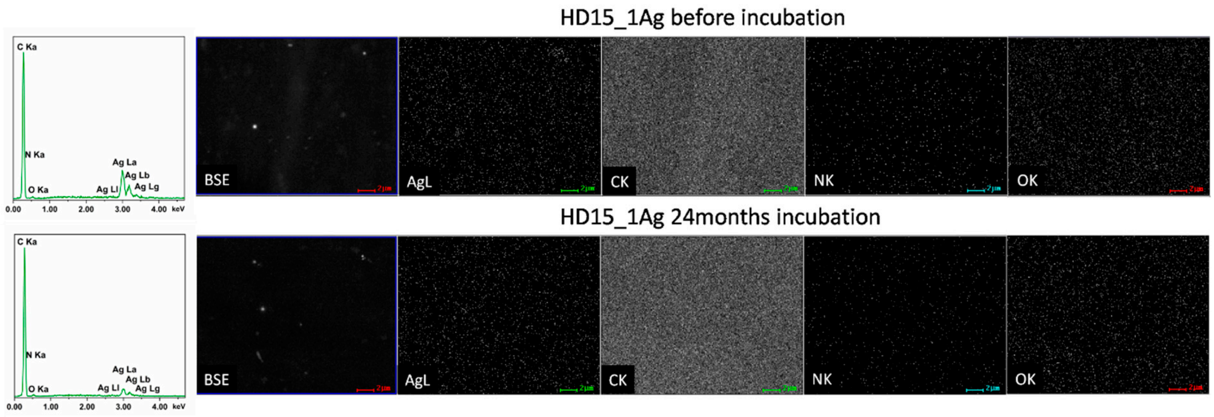

Figure S1. Spectrum and mapping analysis of HD15 polymer containing 1 wt.% of silver nanoparticles (AgNPs) before and 24 months of incubation.
